# Assessment of Aﬂatoxin M1 in human breast milk in Rafsanjan, Iran

**DOI:** 10.18502/cmm.7.1.6177

**Published:** 2021-03

**Authors:** Somayeh Pourtalebi, Seyyed Amin Ayatollahi Mousavi, Zahra Assadollahi, Seyyed Mahdi Mousavi

**Affiliations:** 1 Immunology of Infectious Diseases Research Center, Research Institute of Basic Medical Sciences, Rafsanjan University of Medical Sciences, Rafsanjan, Iran; 2 Department of Microbiology, School of Medicine, Rafsanjan University of Medical Sciences, Rafsanjan, Iran; 3 Medical Mycology and Bacteriology Research Center, Kerman University of Medical Sciences, Kerman, Iran; 4 Department of Epidemiology and Biostatistics, School of Medicine, Rafsanjan University of Medical Sciences, Rafsanjan, Iran; 5 Occupational Environment Research Center, Research Institute of Basic Medical Sciences, Rafsanjan University of Medical Sciences, Rafsanjan, Iran

**Keywords:** Aﬂatoxin M1, Human breast milk, Infant milk, ELISA, Rafsanjan city

## Abstract

**Background and Purpose:**

Aﬂatoxins (AFs) are a group of highly toxic mycotoxins present both in the environment and in foodstuffs. The food of infants should be safe and free of various pollutants, including breast milk mycotoxins. This study aimed to measure the mycotoxin of Aﬂatoxin M1 (AFM1) in human milk samples obtained from lactating mothers living in Rafsanjan city, Iran.

**Materials and Methods:**

In the current cross-sectional study, breast milk samples were collected from 150 lactating mothers in Rafsanjan city from September 2015 to April 2016 using the structured food-frequency questionnaire. The AFM1 was measured by employing enzyme-linked immunosorbent assay specific kits. The statistical analysis was performed in SPSS software (version 16).

**Results:**

The AFM1 was detected in 98 mothers (65%) with a mean concentration of 14.69±8.15 ng/kg, ranging from 5.02 to 41.25 ng/kg. The AFM1 concentration exceeded the tolerable and accepted limit promulgated by the European Union and the USA (25 ng/kg) in only 10 milk samples. Moreover, in 59 milk samples, the AFM1 concentration exceeded the limit recommended by Australia and Switzerland (10 ng/kg).

**Conclusion:**

According to the results of the present study, lactating mothers and their infants are at risk of AFM1 exposure in southern Iran. Accordingly, the examination of AFM1 concentrations in lactating mothers, as a critical postnatal exposure marker of infants to this carcinogenic compound, requires further studies in various seasonal periods and different parts of Iran.

## Introduction

Humans are exposed to numerous toxic chemicals, including carcinogenic substances, in various periods of their life. One of these chemicals is a family of naturally occurring compounds known as Aﬂatoxins (AFs) [ 1 ]. AFs are a group of mycotoxins produced in foods contaminated with various *Aspergillus* molds, especially *A. flavus*, *A. parasiticus*, and *A. nomius* [ 2 ]. Foods are regarded as the main reservoirs for these highly toxic and carcinogenic compounds. Since nutrition is an essential part of human life, the consumption of safe and hygienic foods is a must. 

Given the vulnerability of infants, especially newborns, and the fact that breastfeeding is usually the major path of nutrition during infancy, the quality of breast milk is very important. Evidence in the related literature demonstrates that AFs are found in human biological fluids, including breast milk; hence, the determination of AF levels in body fluids is critical [ 1 ].

The AFs possess genotoxins, teratogens, immu-nosuppressants, and anti-nutritional properties [ 3 ]. To date, four major types of AF have been identified, including aflatoxin B1 (AFB1), aflatoxin B2 (AFB2), aflatoxin G1 (AFG1), and aflatoxin G2 (AFG2) [ 4 ].

Over 18 AFs have been identiﬁed, with AFB1 being the most potent carcinogen among them. In experimental rodents fed with AFB1-contaminated food, this toxin is converted into a secondary hydroxylated metabolite named AFM1, which is subsequently secreted into milk, urine, and feces [ 2 ].

The AFM1 is a form of hydroxylated metabolite excreted in the breast milk of humans and animals exposed to AFB1. This metabolite is reported to be the main excreted product existing in lactating mammals, including the breast milk of women who are exposed to AFB1 through their diets [ 5 , 6 ].

The AFM1 is detectable in milk within 12–24 h from the ﬁrst ingestion of AFB1. In addition, AFM1 is almost resistant to heat, pasteurization, and sterilization, with the toxin becoming detectable when milk is contaminated. The potency of AFM1 toxicity is most often lower than that of its parent compound, i.e. AFB1. The effects of AFM1 including cytotoxic, genotoxic, and carcinogenic effects have been well documented. In this regard, the International Agency for Research on Cancer (IARC) of the World Health Organization has reconsidered carcinogenicity classifications. According to the latest IARC report and criteria, the position of AFM1 has been transferred from Group two to Group one [ 6 , 7 ]. 

Human milk is one of the richest sources of nutrients for infants; therefore, breastfeeding brings enormous psychological, immunological, and economic advantages to both mothers and infants. Although breast milk contains optimally balanced fats, carbohydrates, and proteins, it could also contain toxic chemicals from pollution and other sources. It has been reported that children exposed to AFM1 through either milk or its products could become prone to infectious diseases and underweight their whole life [ 8 ]. 

Several studies have been conducted in different countries on AFM1 levels in the breast milk of lactating mothers and various results have been obtained [ 9 ]. Since pistachio trees are often cultivated in Rafsanjan city in the southeastern province of Kerman, Iran, the AFB1-contaminated pistachio consumption is very likely in lactating mothers who live in this city. Hence, this study aims to examine AFM1 levels (as the transient form of AFB1) in the breast milk of lactating women.

This study primarily aimed to evaluate the presence and levels of AFM1 in lactating mothers in Rafsanjan city, i.e. the pistachio cultivating center of Iran. The present study also aimed to examine possible correlations between AFM1 levels and anthropometric parameters among the studied infants.

## Materials and Methods

### Sample collection

In this cross-sectional study, 150 lactating mothers who had 2-6 months old infants, were enrolled in this study from September 2015 to April 2016. Human milk samples were collected by proportionate stratified sampling from 7 local health centers in urban areas of Rafsanjan. This study was approved by the medical ethics committee of Rafsanjan University of Medical Sciences, Iran (IR.RUMS.REC.1394.63).

Firstly, the nutrition of lactating mothers, including rice, hot dogs [ 10 ], milk, yogurt, cheese, corn, pistachios, and walnuts was checked, and then the milk samples were collected upon their consent. All the fresh milk samples were immediately stored at -20 ºC until the day of analysis which was less than 70 days later (first, 90 samples were collected to perform enzyme-linked immunosorbent assay ). Next, the milk samples were gradually thawed at 4 °C and vigorously mixed up and centrifuged at 10 °C to detect their aﬂatoxin content, being water-soluble [ 6 ]. 

### Analysis of Aﬂatoxin M1 in the samples

Levels of aflatoxin in the milk samples were detected using the competitive ELISA test kit of RIDASCREEN®Aﬂatoxin M1 (R-Biopharm GmBH, Germany). The detection protocol was based on the antigen-antibody reaction that detects the milk levels of AFM1 quantitatively. The mentioned kit was employed in accordance with the manufacturer instructions.

### Preparation of milk samples

The milk samples (10 mL) were subjected to cooling at 10 °C and centrifugation for 10 min at 3500 rpm. The supernatant containing fat was completely discarded, and an aliquot (100 µL per well) of the lower fat-free phase was used for the detection of AFM1.

### Experimental procedure of enzyme-linked immune-sorbent assay

A number of microtiter wells were inserted into a microwell holder for both standards and samples, and a volume of 100 µL of the standards and samples was provided. Next, it was added to the wells and incubated for 30 min at 20–25 °C in the dark. The liquid was removed, and the microwell holder was set in an upside-down position on a filter paper to eliminate the remaining liquid. Afterward, the wells were rinsed twice with 250 µL of the washing buffer. Later on, 100 µL of peroxidase-conjugated AFM1 was added to each well, and the incubation process continued for an additional period of 15 min at 20–25 °C in the dark. 

Once more, the wells were washed three times using 250 µL of the washing buffer. Subsequently, a volume of 100 µL of substrate/chromogen was added to each well, and the plates were incubated again for 15 min at room temperature in the dark. To terminate the reaction, a stop reagent (100 µL) was added to each well, and they were subjected to gentle shaking. In the end, the absorbance rate was read at 450 nm in the ELISA plate reader (ELX-800, Bio-Tek Instruments, USA) against an air blank, within 15 min after the addition of the stop solution.

### Evaluation

According to the experimental protocol, the lowest detection limit was 10 ng/L (10 ppt) and 50 ng/L (50 ppt) for milk and cheese, respectively. The mean values of recovery and coefficient of variation were 95% and 15%, respectively. It must be mentioned that analytical values were not corrected for recovery. All the samples were examined using the RIDAVIN computer program (Art. No.:R1121) provided by R-Biopharm. In addition, statistical data analysis was performed using the RIDA SOFT Win (Art. Nr. Z9999) statistical software.

### Statistical analysis

Both demographic and laboratory examination data were expressed as mean±SD, except for the educational level and occupational status, for which the parameters were shown as number (%). The data obtained from the groups of mothers with contaminated and healthy milk were compared using Pearson’s chi-squared test for the consumed foods. In addition, the one-way ANOVA was used for other continuous variables since the data were normally distributed. 

Binary logistic regression analysis was also performed to compare the contamination prevalence of AFM1 and its related components in the milk of the two groups of mothers. All statistical analyses were two-tailed, and the difference was considered significant for p-values of less than 0.05 (P<0.05). Furthermore, after the raw data collection, relevant data were analyzed in SPSS software (version 16, Chicago, IL, USA).

## Results

According to the results, the mean age of the recruited lactating mothers was 28.99±5.2 years. The mean level of body mass index was high in the examined mothers, and all of them were overweight. The majority of the nursing mothers were housewives who had quitted high school or college. It should be mentioned that all the interviewed mothers were considered healthy. Table 1 shows all the descriptive data for the lactating mothers (n=150) and their infants.

**Table1:** Demographic information of lactating mothers and their infants

Variable		Number	Range	Mean±SD
Age of the mother (year)		150	17- 43	28.99±5.20
Weight of the mother (kg)		150	40 -100	67.80±11.99
Height of the mother (cm)		150	141-173	160.07±5.68
BMI of the mother (kg/m^2^)		150	16 - 40.2	26.40±4.49
Age of the infant (month)		150	1-7	4.65±1.28
Height of the infant at birth (cm)		150	42-55	48.61±2.05
Weight of the infant at birth (kg)		150	2.1- 4.62	3.15±0.39
HC of the infant at birth (cm)	150	31- 42	34.53±1.39
Education status (n (%))	Elementary school		11 (7.3)
	Middle school	14 (9.3)
	High school		65 (43.3)
	University/college		60 (40.0)
Occupational status (n (%))	Housewife		133 (88.7)	
	Employed		17 (11.3)	

BMI=body mass index, HC=Head circumference

The AFM1 was detected in 98 samples out of a total of 150 human breast milk samples, with its median concentration being 14.69±8.15 ng/kg, which ranged from 5.02 to 41.25 ng/kg. Table 2 summarizes the relevant data in this regard. 

**Table2:** Aflatoxin M1 values in breast milk samples (ng/l) of lactating mothers by enzyme-linked immunosorbent assay

Sample	Number of samples	Positive samples	Mean contamination level (ng/L)	
			Range	Mean±SD
Human breast milk	150	98 (% 65.3)	5.02-41.25	14.69±8.15

Contamination rate of the milk of lactating mothers with AFM1 was 68.3%. The AFM1 contamination was more frequent in the milk of mothers who consumed hot dogs (83.3%), peanuts (72.7%), walnuts (70.7%), pistachios (68.1%), cheese (67.9%), yogurt (66.7%), rice (65.8%), milk (58.1%), and corn (28.6%).

Table 3 tabulates the data about breast milk contamination based on food intake in the past 24 h. According to the results of multivariate logistic regression, mothers who consumed walnuts exhibited a higher possibility of AFM1 contamination (2.278) in their milk, compared to mothers who did not consume nuts. In addition, the ratio was statistically significant for walnuts (P=0.049), while other ratios were not statistically significant (P>0.05) ( Table 4).

**Table3:** Comparison of daily food consumption in mothers with contaminated and healthy milk in Rafsanjan

Food	Aflatoxin M1		*p-value*
	Mothers with contaminated milk (%)	Mothers with safe BM (%)	
Rice	79 (65.8)	41 (34.2)	0.797
Hot dog	10 (83.3)	2 (16.7)	0.172
Milk	25 (58.1)	18 (41.9)	0.241
Yogurt	40 (66.7)	20 (33.3)	0.779
Cheese	57 (67.9)	27 (32.1)	0.464
Corn	2 (28.6)	5 (71.4)	*0.036
Pistachio	77 (68.1)	36 (31.9)	0.207
Peanut	8 (72.7)	3 (27.3)	0.592
Walnut	41 (70.7)	17 (29.3)	0.274

BM=Breast milk,

* =significant difference with the safe BM group

**Table4:** Results of Multivariate analysis of the correlation between the consumption of selected foods and AFM1 contamination of breast milk correlations represented regarding odds ratio, coefficient (B), and related *p-value*

Food	Aflatoxin M1		
	Odd ratios	Coefficient (B)	*p-value*
Rice	1.178	0.164	0.729
Hot dog	4.005	1.388	0.100
Pasteurized milk	6.671	1.898	0.121
Sterilized milk	1.045	0.044	0.917
Bulk milk	0.518	-0.657	0.292
Yogurt	1.052	0.051	0.898
Cheese	1.33	0.285	0.464
Corn	0.079	-2.534	0. 140
Pistachio	2.334	0.848	0.060
Peanut	1.235	0.211	0.778
Walnut	2.278	0.823	0.049

*=significant

## Discussion

Breast milk could be contaminated by the consumption of contaminated nutrients at various levels [ 11 , 12 ]. The early and immediate exposure of infants to aflatoxin has been proved to cause immunosuppression, growth inhabitation and impairment, as well as the state of being underweight. Therefore, the breast milk containing AFM1 should be considered a potential health-threatening toxin able to endanger the health and life of the infants.

Regulations on the acceptable concentrations of AFM1 in milk and infant foods vary in different countries. The European Communities and Codex Alimentarius have prescribed a limit of 25 ng/kg of AFM1 in breast milk. Similarly, the US regulations also prescribed the same limit of AFM1 in breast milk. In Australia and Switzerland, the maximum level of AFM1 in infant milk is 10 ng/kg [ 12 , 13 ]. 

In this study, the concentration of AFM1 was measured in the breast milk of 150 lactating mothers in Rafsanjan. Based on the results, the mean contamination of AFM1 in 65.3 % of milk samples was 14.69±8.15 ng/L, ranging from 5.02 ng/L to 41.25 ng/L. The mean concentration values of AFM1 in the breast milk samples of lactating mothers in Colombia [ 14 ], Turkey [ 8 ], and Cyprus [ 15 ] were 5.2, 3.01, and 7.84 ng/L, respectively. 

In this study, the mean concentration of AFM1 in the breast milk samples of the studied mothers was lower than that of the samples in the previous research [ 12 , 13 , 16 ]. This could be due to a variety of factors, including several environmental and seasonal changes as well as the conditions of harvesting and storage of nuts before marketing which is an effective factor in AFM1 contamination levels in breast milk [ 12 , 13 , 16 ].

Turkish investigators reported that the minimum and maximum concentration levels of AFM1 in the breast milk of Turkish lactating mothers were 60.90 ng/L and 299.99 ng/L, respectively [ 1 ]. Other studies have reported an AFM1 concentration of 71 ng/L in Australia, 664 ng/L in Thailand [ 16 ], and 401 ng/L in Sudan [ 17 ]. These high levels of concentration of AFM1 are due to the conditions of warmth and humidity, different diets, and the possibility of contamination of imported foods. Since our sampling was carried out during autumn and winter, the weather conditions were not effective in the results of this study.

Mahdavi et al. examined AFM1 contamination in the breast milk of lactating mothers in both urban and rural regions of Tabriz, in northeastern Iran. In their study, they reported the level of AFM1 contamination at 22% in rural areas (with the mean concentration of 6.96±0.94 ng/L); however, they reported no AFM1-positive samples in the urban areas of Tabriz [ 18 ]. In another study performed in Hamadan, Iran, the occurrence rate and level of breast milk AFM1 were considerably higher in rural regions, compared to urban areas. This might have been due to the different dietary habits and socio-economic status of the participants [ 19 ].

 In another study recently conducted in Isfahan, Iran, AFM1 was detected in only one out of 80 breast milk samples (1.25%), with a concentration of 6.88 ng/L [ 20 ]. Breast milk AFM1 levels were also examined in 85 lactating mothers in Ilam province, Iran. Based on the results of the aforementioned study, AFM1 was detected in all the samples with a mean concentration of 5.91±2.031 ng/L, ranging from 2 to 10 ng/L [ 2 ]. Afshar et al. conducted a study on 136 samples in Sari, Iran, and found only one AFM1-positive sample (0.7%) with a concentration of 20 ng/L (0.73%) [ 10 ]. 

Sadeghi et al. also reported that 157 out of 160 breast milk samples collected from lactating mothers were contaminated with AFM1 in Tehran province, Iran, ranging from 0.3 to 26.7 ng/L, with a mean concentration of 8.2±5.1 ng/L [ 6 ]. Variations in the results could have been caused by several factors, including environmental and socio-economic factors, employed analytical techniques, food storage methods, and dietary habits.

In the present study, the mean concentration of breast milk AFM1 was 14.69±8.15ng/L in Rafsanjan city, which was lower than the tolerable limit announced by the EU and US regulations. However, it was higher than the limit declared by Australia and Switzerland. This is the first study to have investigated AFM1 contamination in lactating mothers in southern Iran. Evaluation of the breast milk of mothers in Rafsanjan health centers revealed unexpected levels of AFM1 which indicates insignificant exposure of mothers to aflatoxin. It is noticeable that the contamination of milk and dairy products with AFM1 is endemic in Iran [ 21 , 22 ].

Although several studies have been conducted on AFM1 in different parts of Iran, the contamination of diets with AFM1 is yet to be explored; hence, the potential risk of contamination of biological fluids with this toxin is an essential concern that requires further study.

The mentioned levels of AFM1 contamination in lactating mothers in Rafsanjan city could have been due to the consumption of pistachios and other nuts, as contaminated foodstuffs, which are popular in this city of Iran. A considerable portion of pistachio nuts is cultivated in Rafsanjan city that is known as the major producer of pistachios worldwide. Although nuts are consumed by humans, all other mammals, including cows, sheep, and goats eat tree nuts as well as other parts of trees, including leaves, bark, and nutshell. Therefore, some types of AFM1 contamination could be due to the contamination of mammalian milk and meat through consuming contaminated nuts.

Hence, we expected high levels of AFM1 in the milk of mothers who consumed pistachios. Unlike previous research performed on human breast milk [ 6 , 17 ], in this study, the consumption of various nuts (e.g., pistachio, peanut, and walnut), nutrients (rice, yogurt, cheese, milk, and corn), and fast food (e.g., hot dogs) was evaluated.

Despite our expectations, mothers who had eaten hot dogs, peanuts, and walnuts showed higher levels of AFM1 in their milk than those who had eaten pistachios ( Table 3). Similarly, the results of the study conducted by Jafarian Dehkordi et al. demonstrated that only one out of 80 mothers who had consumed hot dogs in the last 24 h prior to sampling was contaminated with AFM1 [ 22 ]. 

The results of this study demonstrated that only the relationship between contamination with aflatoxin and corn consumption was statistically significant; however, other findings were also important. For example, 83% of mothers who consumed hot dogs were contaminated with aflatoxin ( Table 3).

Therefore, currently, the levels of this mycotoxin in the milk of Iranian lactating mothers may not be risky; however, these results do not exclude the remarkable exposure of children to AFM1 that could adversely aﬀect their health. Hence, it is important that AFM1 be routinely monitored as a quality control parameter of foods.

## Conclusion

Results of the present study revealed the possibility of the presence of AFM1 in breast milk samples obtained from lactating mothers in Rafsanjan city, Iran. The AFM1 was detected in 65.3% of the breast milk samples studied in this research. This contamination could have been due to the different dietary patterns of the lactating mothers. According to the results, dietary habits with more portions of hot dogs and nuts increase the risk of AFM1 contamination in breast milk. Further studies should be conducted in different regions of Iran to examine the overall association between AFM occurrence and dietary factors, as well as the risk of infant exposure to this mycotoxin.

Apart from human breast milk, infants are at the potential risk of exposure to AFM1 by meat, eggs, milk, and other edible products from animals consuming AF-contaminated nutrients. Therefore, there is an urgent need for continuous monitoring of the level of AFM1 contamination in lactating mothers to ensure the protection of the infant. 
